# Restrictions on the Sale of Poisonous Drugs

**Published:** 1919-03-01

**Authors:** 


### RESTRICTIONS ON THE SALE OF POISONOUS DRUGS.

### How the Law Stands.

The prominence given in the newspapers to certain recent cases in which death ensued apparently in consequence of a perverted drug habit suggests the inquiry as to how the law at present stands and whether it is practicable by amendment of it to forbid or lessen facilities for indulging the drug habit. The general law as to the sale and distribution of poisonous drugs in Great Britain is contained in the *Arsenic Act of* 1851 and in the *Pharmacy Act of* 1868 as amended by the *Poisons and Pharmacy Act of* 1908. The provisions of these statutes will be generally familiar to our readers and we need not detail them: what we propose to do, however, is to call attention to the remarkable manner in which these measures have been strengthened by the emergency legislation of the recent war period.

### Importation without Licence Prohibited.

It having been found necessary during the progress of the war to restrict further than was provided by the ordinary law, the supply and distribution of cocaine and opium, this step was taken by the machinery of statutory order. By Order in Council dated July 28, 1916, made under Section 43 of the *Customs Consolidation Act*, 1876, the importation of cocaine or opium into the United Kingdom without a licence from one of the principal Secretaries of State, was prohibited and for the purposes of the Order any preparation or mixture made from or with cocaine and containing one part in a thousand of the drug was to be considered to be cocaine. For any offence against the Order an offender is liable to a penalty of either treble the value of the goods or £100, and he may be detained or proceeded against by summons.

### A Summary Offence under "Dora."

Intoxicants have been further dealt with by the Defence of the Realm Regulations 40A, which makes it a summary offence under the regulations for any one to supply to any member of His Majesty's Forces who is undergoing hospital treatment otherwise than under doctor's orders in connection with such treatment. This regulation does not define "intoxicant," which is defined by the Regulation 40 to include any sedative, narcotic, or stimulant, drug, or preparation. And under 40B if any person sells, gives, procures or supplies, or offers to sell, give, procure or supply cocaine or opium to or for any person other than an authorised person, he is guilty of a summary offence under the regulations unless he complies with conditions laid down in the regulations.

### Who is an Authorised Person.

The "authorised person," supply to whom is not restricted, means a duly qualified medical practitioner, a registered dentist, a registered veterinary surgeon, a person, firm, or body corporate entitled to carry on the business of chemist and druggist under the Pharmacy Acts, a licenciate of the Apothecaries' Hall in Ireland, a person holding a general or special permit from the Home Secretary to purchase the drug in question, or an occupier of a factory or workshop who holds a general permit. To any other person cocaine can only be supplied by a person duly authorised to dispense and on a prescription in writing of a duly qualified medical practitioner. The prescription must be marked with the words "not to be repeated," and must specify the total amount of cocaine to be supplied. The name and business address of the person dispensing with the date on which it is dispensed must be marked on the prescription, and cocaine must not be again supplied on the same prescription unless on fresh directions duly endorsed and signed by the practitioner by whom it was originally issued. The person dispensing must keep on his premises a book in which he enters particulars in the prescribed form of the drug supplied, and this book must be open to inspection by any authorised person appointed for the purpose by the Home Secretary. A dealer in either cocaine or opium is also required to keep a record in the prescribed form of all his dealings in these drugs, and the record is similarly to be kept open for inspection.

The commoner form of the abuse of opium is specially provided against. It is a summary offence under the Defence of the Realm Regulations for any person to prepare opium for smoking or to deal in or have in his possession any opium so prepared, or to sell or otherwise supply opium to or for any person in the United Kingdom other than an authorised person so above defined. And as regards both drugs no person other than an authorised person or a person duly licensed to import may have either in his possession.

The records required to be kept by every person who deals in any way in cocaine or opium are to be separate for each drug, and must contain the following particulars: (1) Date of sale; (2) name of person or body to whom sold; (3) address of person or body to whom sold; (4) authority of person or body to purchase; (5) amount of opium or cocaine sold; (7) (for cocaine only) when sale is on a prescription, the ingredients of the prescription.

We are indebted to our contemporary *The Justice of the Peace* for a review of the whole legislation on this subject, from which the foregoing account has been largely extracted, and would conclude by quoting the general conclusion at which the reviewer arrives: "Drugs taken as part of an ordered and skilled regimen, so far from being injurious, are in general beneficial to the human frame, and finally that it is undoubted that the events on the publication of which a change in the law is called for would most likely not have been revealed at all, and consequently would have been without their influence on public opinion but for the operation of the law at present existing to restrain a habit which any law, however stringent, would probably be found ineffective to wholly eradicate."

